# Muffins enriched with dietary fiber from kimchi by‐product: Baking properties, physical–chemical properties, and consumer acceptance

**DOI:** 10.1002/fsn3.1020

**Published:** 2019-04-10

**Authors:** Yena Heo, Min‐Joo Kim, Jo‐Won Lee, BoKyung Moon

**Affiliations:** ^1^ Department of Food and Nutrition Chung‐Ang University Anseong‐si Korea

**Keywords:** Chinese cabbage, dietary fiber, kimchi by‐product, muffin, quality characteristics

## Abstract

This study aimed to evaluate the effects of dietary fiber from Chinese cabbage outer‐leaf powder, which is a main by‐product of kimchi, on the quality, texture properties, and sensory evaluation of muffins. The kimchi by‐product powder (KBP, 36.2% dietary fiber) was added at 1%–4% dietary fiber content, by replacing wheat flour (w/w basis). The physico‐chemical and sensory properties of the baked muffins were measured. The height and volume of the muffins decreased with the addition of KBP. Increasing the KBP content resulted in increased hardness and reduced chewiness. No significant difference was observed in the overall acceptance among the muffins, up to the 2% added dietary fiber group, and the positive effect of the incorporated KBP was also confirmed in the sensory evaluation. These results indicate that it is possible to produce functional muffins with increased dietary fiber content by adding KBP in place of flour.

## 1 INTRODUCTION

Muffins are a favorite bakery product among consumers, owing to the soft texture and good taste. However, muffins have a low nutrient density, as they are high in sugar and fat and low in dietary fiber (Romjaun & Prakash, 2013 and Cho & Kim, 2014).

Given the well‐known advantages of a healthy diet, the food industry has responded by developing new foods with health‐promoting characteristics. In particular, dietary fiber is indispensable for health maintenance (Lebesi & Tzia, 2011). Dietary fiber intake is important in the prevention of diseases such as hypercholesterolemia, colon cancer, coronary heart disease, and hypertension (Romjaun & Prakash, 2013 and Lebesi & Tzia, 2011). Several attempts have been made to replace wheat flour with various dietary fibers, such as ascidian tunic (Yook et al., 2000), banana peel (Sodchit, Tochampa, Kongbangkerd, & Singanusong, 2013), potato skin (Arora & Camire, 1994), oat bran, rice bran, or barley fiber fractions (Hudson, Chiu, & Knuckles, 1992), and peach fiber (Grigelmo‐Miguel, Shela, & Olga, 1999). The nutritional and functional properties of various dietary fibers have also been examined (Lario et al., 2004).

Kimchi, a traditional Korean fermented food composed mainly of Chinese cabbage, plays a significant role in the diet and nutrition of Koreans, and sales of kimchi exceed 130 million dollars per year in Korea (Kim & Chun, 2005). As the consumption of kimchi increases, so does the waste derived from the kimchi trimming process (Choi & Park, 2003). This waste mainly consists of the outer leaves of Chinese cabbage, which are usually discarded or used as fertilizer or animal feed (Jongaroontaprangsee et al., 2007) but also causing environmental and economic issues due to their rapid decay in the natural environment and high processing costs (Liu, Ko, Kim, & Kim, 2012). However, the outer leaves of Chinese cabbage have high fiber content, making them a good source of dietary fiber (Lario et al., 2004). Numerous studies have reported the application of waste products as a source of dietary fiber, including banana peels (Sodchit et al., 2013), lemon juice (Lario et al., 2004), apple pomace (Sudha, Baskaran, & Leelavathi, 2007), white cabbage (Gul, Yanik, & Acun, 2013), orange (Miguel & Bellose, 1999), and pineapple (Nigam, 1999) waste.

This study investigated the possibility of incorporating kimchi by‐product, as a source of dietary fiber, into a bakery product. For this purpose, muffins were formulated with increased dietary fiber (0%, 1%, 2%, 3%, and 4%), by adding kimchi by‐product powder (KBP) in replacement of wheat flour (w/w basis). The pasting properties, color, texture, dietary fiber content, and sensory attributes of the muffins were evaluated.

## 2 MATERIALS AND METHODS

Commercial soft flour (CJ Co., Seoul, South Korea), sugar (CJ Co.), butter (Seoulmilk ICA, Seoul, South Korea), skim milk powder, vanilla powder, baking powder, eggs, and milk were purchased from a local market in Korea. Kimchi by‐products were obtained from a kimchi factory, washed to remove soil and dirt, drained, and dried at 70°C for 24 hr, using a hot air drier (Lequip, Hwaseong, Gyeonggi‐do, Korea). After cooling to room temperature (24°C), the material was ground and strained through a 50‐mesh sieve (297 μm). The powder was stored in a polyethylene bag at −18°C. A total dietary fiber (TDF) assay kit (K‐TDFR) was obtained from Megazyme International (Bray Co., Wicklow, Ireland). MES (2‐*N*‐morpholino ethanesulfonic acid) hydrate and Trizma base were purchased from Sigma‐Aldrich (St. Louis, MO, USA).

### 2.1 Muffin preparation

Muffin formulations are presented in Table 1. The amount of KBP incorporated into muffins corresponded to 1%–4% dietary fiber. KBP consists of 36.2% TDF; therefore, 4.14, 8.29, 12.43, or 16.57 g KBP was added, replacing an equal weight of wheat flour. Butter and sugar were mixed using a hand mixer (CONCEPT‐190L, Penta Korea, Seoul, South Korea) for 3 min, and an egg was added, with four times mixing for 5 min at speed 4. After incorporating the dry ingredients (flour, baking powder, vanilla powder, skim milk powder, and KBP), by mixing for 1 min (speed 1), the batter was blended with the milk for 2 min (speed 2), divided (55 g aliquots) into paper molds, and baked at 180°C in a preheated electric oven (FDO‐7102, Daeyung, Seoul, South Korea) for 25 min. The muffins were left to cool on a rack for 1 hr, to avoid accumulation of condensation (Goswami, Gupta, Mridula, Sharma, & Tyagi, 2015).

**Table 1:** Formulation of muffins containing kimchi by‐product powder (KBP)

Ingredient (g)	Control	KBP 1^a^	KBP 2	KBP 3	KBP 4
Wheat flour	150.00	145.86	141.71	137.57	133.43
KBP	0.00	4.14	8.29	12.43	16.57
Milk	120	120	120	120	120
Sugar	110	110	110	110	110
Butter	70	70	70	70	70
Egg	40	40	40	40	40
Skim milk powder	15	15	15	15	15
Baking powder	3.5	3.5	3.5	3.5	3.5
Vanilla powder	1.5	1.5	1.5	1.5	1.5

a KBP 1, 2, 3, and 4: dietary fiber in KBP replaced 1%, 2%, 3%, and 4% of the flour, respectively.

### 2.2 Composition of the muffins

Moisture, protein, crude fat, and ash of muffins were analyzed according to the Association of Official Analytical Chemists [AOAC] methods (2012). Total carbohydrate content was determined by difference. TDF contents were measured by method 991.43 (AOAC, 2012). One gram of sample in MES–Tris buffer (adjusted to pH 8.2) was placed in a beaker. After α‐amylase (50 μl) was added, the samples were incubated for 30 min (100°C) and then cooled to room temperature. After the addition of protease (100 μl), the samples were incubated at 60°C for 30 min. Once cooled, 5 ml HCl (0.561 N, pH 4.5 ± 0.2) and 200 ml amyloglucosidase were added, followed by incubation at 60°C for 30 min. After adding 200 ml ethanol (95%), the mixture was incubated at room temperature for 60 min. The residue and slurry were washed twice each with 15 ml of 78% and 95% ethanol, respectively. The final slurry was washed with 15 ml of 78% and 95% ethanol, respectively, and the TDF was measured. Half of the final slurry was used for crude protein analysis (Kjeldahl), and the other half was analyzed for ash by burning at 550°C for 5 hr.

### 2.3 Microstructure of the muffin by scanning electron microscopy

The microstructure of the muffin crumbs was examined using a scanning electron microscope (Hitachi‐S‐3400N, Hitachi Ltd., Tokyo, Japan). In order to measure, the muffin crumb was cut into 10 mm cube and was sputter‐coated with gold after freeze‐drying. Each sample was observed at an accelerating voltage of 5 kV. The scanning electron microscopy (SEM) images of the muffins were acquired at 1,500× magnification (Manaf, Othman, Harith, & Ishak, 2017).

### 2.4 Physical characteristics of the muffins

The height, volume, symmetry, and uniformity of muffins containing KBP were measured by using a caliper, and the weight and baking loss rate were measured on an electronic scale (HS 2140A, Hansung, Seoul, South Korea), using the modified American Association of Cereal Chemists [AACC] method 10‐91 (AACC, 2000).

The baking loss rate was calculated by the following equation:

(1) $$\text{Baking loss rate}\left(\%\right)=\frac{\left(\text{Batter weight}-\text{Muffin weight}\right)\left(\text{g}\right)}{\text{Batter weight}\left(\text{g}\right)}\times 100$$

Images of the muffins were photographed against a white background, using a digital camera.

### 2.5 Color measurement

The colors of muffin crusts and crumbs were measured using an Ultrascan PRO Colorimeter (Hunter Lab, VA, USA), pre‐calibrated with a standard white background tile (L* = 99.56, a* = −0.17, b* = −0.21). The color was measured 1 hr after baking.

### 2.6 Texture profile

The texture of the muffins was analyzed using a texture analyzer (TA.HDi/500, Stable Micro Systems, UK) equipped with an SMS p/36R probe (Stable Micro Systems) following the method of Alvarez Herranz Jiménez and Canet (2017). To measure the texture of complete crumb lower half, samples were cut horizontally at the height of the mold; the upper half was discarded, and the 2.5‐cm‐high lower half was removed from the mold. The texture instrument parameters were as follows: 2 mm/s pretest and test speed, 4 mm/s post‐test speed, 5 g trigger force, 30 mm distance, and 40% strain. The hardness, adhesiveness, springiness, cohesiveness, chewiness, and resilience were measured.

### 2.7 Sensory properties

The appearance, color, texture, flavor acceptance for muffin, and overall acceptance for quality were evaluated by 120 panelists using a 7‐point hedonic rating scale (1 = lowest acceptance; 7 = highest acceptance). The panelists aged between 20 and 40 years (25 ± 6 years), and 10% of the panelists was male and 90% of them was female. The muffins were removed from the oven, cooled at room temperature (24°C) for 1 hr, cut into four pieces, and randomly assigned a coded 3‐digit number. One piece of each muffin was provided to the panelists, along with a cup of water to neutralize the taste between each sample.

### 2.8 Antioxidant property of the muffins

The 2,2‐diphenyl‐1‐picrylhydrazyl (DPPH) radical scavenging activity of the muffins was determined according to the method of Kim et al. (2012) with some modifications for filtration. Ethanolic extracts of muffins were prepared by mixing 5 g of sample with 45 ml of 70% ethanol on a shaking incubator at 37°C for 24 hr. The mixture was centrifuged (7,041 *g*) at 4°C for 10 min. An aliquot (0.1 ml) of the resultant supernatant was mixed with 0.1 ml of an ethanolic solution of DPPH (0.1 mM, 80%) and incubated at 25°C. After 30 min, the absorbance of the mixture was read at 517 nm on a microplate reader (Eon; BioTek Ltd., Winooski, VT, USA).

### 2.9 Statistical analysis

Quantitative data are expressed as mean ± standard deviation (*SD*) of triplicate measurements. The data, which represent at least triplicate measurements, were analyzed using SAS version 8.0 for Windows (SAS Institute, Inc., Cary, NC, USA) with one‐way analyses of variance (ANOVA). Duncan's multiple range test at *p* < 0.05 was used to define significance.

## 3 RESULTS AND DISCUSSION

### 3.1 Composition, appearance, microstructure, and antioxidant property of the muffins

Table 2 shows the proximate composition of the muffins. The KBP‐containing muffins had a higher TDF content (8.64%–12.73%) than the control (6.71%). As expected, the dietary fiber level increased with the increasing addition of KBP. According to our previous study (Lee et al., 2016), Chinese cabbage outer‐leaf powder, which had a high TDF content, was reported as a good source of functional ingredients. KBP‐added muffins showed higher moisture contents than the control. The higher the KBP content, the higher the moisture content, owing to the water‐holding capacity of the dietary fiber, as previously noted by Kim et al. (2012). Similar results were also demonstrated in studies of muffins with peach fiber (Grigelmo‐Miguel et al., 1999), waxy whole‐wheat flour (Acosta, Cavender, & William, 2011), and cocoa fiber (Martinez‐Cervera, Salvador, Muguerza, Moulay, & Fiszman, 2011), added as sources of dietary fiber. Based on these results, KBP was considered as a promising source of dietary fiber that can be added to muffins. There were no significant differences in water activity among the samples. Protein and ash increased significantly with the addition of KBP. According to Zin, Robert, and Ishak (2014), the inorganic components in food are related to the ash content. Therefore, KBP was considered to have the capability to improve the mineral contents of muffins. The carbohydrate content and the energy value of samples with KBP were significantly lower than those of the control because the carbohydrate content was reduced by the replacement of wheat flour with KBP.

**Table 2:** Composition and antioxidant property of muffins containing kimchi by‐product powder (KBP)

Sample^2^	Moisture (%)	Protein (%)	Fat (%)	Ash (%)	Carbohydrate (%)	Dietary fiber (%)	Energy (kcal/100 g)	Water activity	DPPH antioxidant activity (%)
Control	20.38 ± 0.03^d^ ^1^	6.68 ± 0.01^c^	14.83 ± 0.20^b^	1.13 ± 0.01^e^	57.00 ± 0.25^a^	6.71 ± 1.40^e^	374.76 ± 0.34^a^	0.94 ± 0.01	24.87 ± 0.12^e^
KBP 1	21.71 ± 0.04^c^	6.71 ± 0.07^c^	15.33 ± 0.16^a^	1.31 ± 0.03^d^	54.98 ± 0.22^b^	8.64 ± 1.80^d^	363.54 ± 0.35^b^	0.93 ± 0.03	28.04 ± 0.32^d^
KBP 2	21.78 ± 0.20^c^	6.97 ± 0.01^b^	14.90 ± 0.21^b^	1.57 ± 0.03^c^	54.79 ± 0.45^b^	9.56 ± 0.40^c^	361.98 ± 0.35^b^	0.94 ± 0.01	30.69 ± 0.10^c^
KBP 3	22.42 ± 0.10^b^	6.89 ± 0.04^b^	15.13 ± 0.12^ab^	1.71 ± 0.01^b^	53.84 ± 0.15^c^	10.83 ± 3.20^b^	357.46 ± 0.66^c^	0.94 ± 0.04	34.66 ± 0.15^b^
KBP 4	23.90 ± 0.05^a^	7.09 ± 0.02^a^	15.33 ± 0.12^a^	1.94 ± 0.01^a^	51.75 ± 0.16^d^	12.73 ± 1.00^a^	347.82 ± 0.57^d^	0.93 ± 0.03	37.56 ± 0.23^a^

Note

A different superscript letter in the same column indicates statistical difference (*p < *0.05).

^1^ KBP 1, 2, 3, and 4: dietary fiber in KBP replaced 1%, 2%, 3%, and 4% of the flour, respectively.

^2^ All data represented as mean ± standard deviation (*n* = 3).

**Figure 1:**
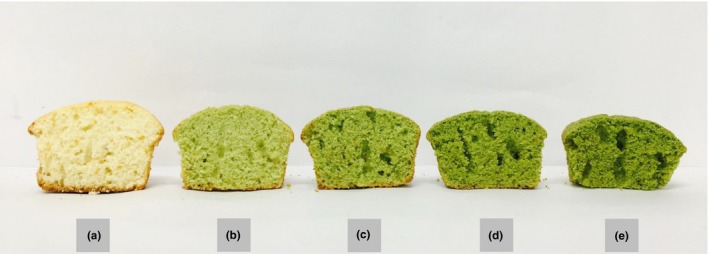
Vertical sections of muffins containing different amounts of dietary fiber in KBP. (a) control muffin without KBP; (b) muffin with 1% dietary fiber; (c) muffin with 2% dietary fiber; (d) muffin with 3% dietary fiber; and (e) muffin with 4% dietary fiber

Figure 1 illustrates the cross‐sections of vertically cut muffins containing different amounts of KBP. The addition of KBP inhibited the formation of air cells, resulting in a dense and voluminous matrix. In Figure 1d,e, the gluten structure of the KBP‐enriched muffins, which retained the air cells, collapsed and appeared to form a tunnel. These results were similar to those observed by Jung, Kim, and Chung (2005) who studied muffins with added corn bran fiber.

**Figure 2:**
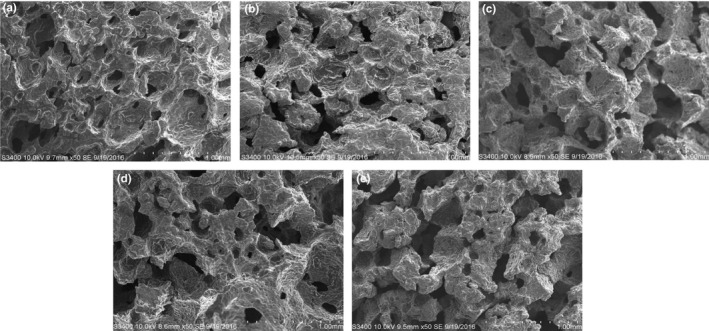
Scanning electron microscopy micrographs of muffin crumb containing kimchi by‐product powder. (a) control muffin without KBP; (b) muffin with 1% dietary fiber; (c) muffin with 2% dietary fiber; (d) muffin with 3% dietary fiber; (e) muffin with 4% dietary fiber

According to the SEM images of the muffin crumbs (Figure 2), the control contained small and uniform air pockets while muffins incorporated with KBP exhibited an irregular, dense structure with a decreased number of air pockets. These results might be caused by the decreased gluten present, due to the replacement of gluten by KBP, which interfered with optimal gluten network formation. This phenomenon also affected the volume and height of the muffins. In corroboration with this finding, Lee et al. (2015) reported a reduced volume and height of muffins when flour was replaced by other ingredients.

The results for DPPH radical scavenging activity of muffins are shown in Table 2. DPPH radical scavenging activity of KBP was measured as 41.71%. Control had the lowest antioxidant activity value, and as the KBP content increased from 0% to 4%, the DPPH radical scavenging activity of muffins gradually increased (24.87%–37.56%). Seong, Hwang, and Chung (2016) described the Chinese cabbage outer leaves as having stronger antioxidant activities than other leaf parts. From the dietary fiber results, the higher the KBP content, the higher the dietary fiber content of the muffins. Elleuch et al. (2011) reported that the antioxidant activities of dietary fibers could improve the oxidative stability and extend the shelf life of food products. Therefore, it might be deduced that the addition of KBP to baked products, such as muffins, which are high in fat, could contribute to the freshness of the products during storage.

### 3.2 Physical characteristics of the muffins

From the baking properties of the muffins (Table 3), the control muffins had the highest volume, and the height and volume decreased significantly (*p < *0.05) with the addition of KBP. Likewise, Walker, Tseng, Cavender, Ross, and Zhao (2014) described a decreased muffin volume with the addition of wine grape pomace, and Martinez‐Cervera et al. (2011) observed a lowered muffin height with increasing cocoa fiber. Volume and form are important characteristics of baked goods (Peressini & Sensidoni, 2009). The decreases in final volume and height of the bakery product caused by the addition of fiber (Peressini & Sensidoni, 2009) might be explained by the dilution of the gluten. Uniformity and symmetry were not significantly different among the samples (Table 4). The weight of the muffins increased significantly with the addition of KBP, and the baking loss rate decreased accordingly. Bagheri and Seyedein (2011) documented similar results in a dough with rice bran fiber.

**Table 3:** Physical properties of muffins containing kimchi by‐product powder (KBP)

Sample^1^	Height (cm)	Weight (g)	Loss rate (%)	Volume	Symmetry	Uniformity
Control	4.83 ± 0.08^a^ ^2^	48.74 ± 0.11^c^	11.38 ± 0.21^a^	13.83 ± 0.23^a^	0.67 ± 0.15	0.10 ± 0.02
KBP 1	4.75 ± 0.05b	49.22 ± 0.24^b^	10.51 ± 0.43^b^	13.60 ± 0.11^b^	0.65 ± 0.12	0.08 ± 0.04
KBP 2	4.65 ± 0.05^c^	49.28 ± 0.28^ab^	10.40 ± 0.50^bc^	13.22 ± 0.16^c^	0.73 ± 0.10	0.07 ± 0.05
KBP 3	4.50 ± 0.06^d^	49.44 ± 0.11^ab^	10.11 ± 0.21^bc^	12.93 ± 0.22^d^	0.57 ± 0.15	0.07 ± 0.05
KBP 4	4.45 ± 0.05^d^	49.52 ± 0.15^a^	9.96 ± 0.27^c^	12.62 ± 0.18^e^	0.73 ± 0.14	0.07 ± 0.05

Note

A different superscript letter in the same column indicates statistical difference (*p < *0.05).

^1^ KBP 1, 2, 3, and 4: dietary fiber in KBP replaced 1%, 2%, 3%, and 4% of the flour, respectively.

^2^ All data represented as mean ± standard deviation (*n* = 3).

**Table 4:** Color and texture profiles of muffins containing kimchi by‐product powder (KBP)

Parameter	Control	KBP 1^1^	KBP 2	KBP 3	KBP 4
Crust color					
L*	69.32 ± 2.26^a^ ^2^	59.32 ± 3.64^b^	57.27 ± 1.14^b^	54.49 ± 1.38^c^	53.68 ± 0.75^c^
a*	12.44 ± 1.56^a^	2.10 ± 0.65^b^	1.30 ± 0.49^bc^	0.93 ± 0.28^c^	0.41 ± 0.94^c^
b*	34.95 ± 0.91^a^	26.53 ± 2.71^b^	25.76 ± 1.38^b^	22.12 ± 0.74^c^	21.18 ± 0.76^c^
Browning index	86.83 ± 4.12^a^	65.41 ± 4.66^b^	64.97 ± 4.30^b^	57.20 ± 3.29^c^	54.54 ± 2.31^c^
Crumb color					
L*	21.18 ± 0.76^c^	69.98 ± 0.51^b^	63.00 ± 0.56^c^	58.50 ± 0.75^d^	55.81 ± 0.79^e^
a*	1.85 ± 0.22^a^	−3.46 ± 0.26^b^	−4.39 ± 0.36^c^	−4.52 ± 0.20^c^	−4.53 ± 0.29^c^
b*	24.37 ± 0.48^a^	30.29 ± 0.34^a^	30.08 ± 0.75^b^	27.62 ± 0.60^c^	25.85 ± 0.77^d^
Texture profile					
Hardness (g)	412.13 ± 24.17^b^	444.23 ± 52.89^ab^	453.37 ± 25.46^ab^	486.55 ± 65.45^ab^	491.81 ± 72.04^a^
Adhesiveness (g/s)	−0.52 ± 0.37^a^	−1.09 ± 1.19^a^	−1.49 ± 1.03^a^	−1.54 ± 1.07^a^	−1.88 ± 1.64^a^
Springiness	0.85 ± 0.01^a^	0.82 ± 0.01^b^	0.79 ± 0.01^c^	0.79 ± 0.01^c^	0.75 ± 0.03^d^
Cohesiveness	0.59 ± 0.05^a^	0.54 ± 0.01^ab^	0.50 ± 0.03^bc^	0.51 ± 0.04^bc^	0.49 ± 0.02^c^
Chewiness	275.00 ± 18.65^a^	212.23 ± 17.32^b^	180.97 ± 18.97^b^	175.03 ± 33.14^bc^	140.01 ± 31.94^c^
Resilience	0.26 ± 0.02^a^	0.23 ± 0.01^b^	0.21 ± 0.01^b^	0.21 ± 0.02^b^	0.18 ± 0.02^c^

Note

A different superscript letter in the same column indicates statistical difference (*p < *0.05).

^1^ KBP 1, 2, 3, and 4: dietary fiber in KBP replaced 1%, 2%, 3%, and 4% of the flour, respectively.

^2^ All data represented as mean ± standard deviation (*n* = 3).

The crust and crumb color values of the muffins (Table 4) revealed a significant decrease in L*, a*, and b* values of both the crust and crumbs, by adding KBP. The same trend was found by Sudha et al. (2007). As the amount of KBP increased, the crumb color changed more than the crust color. According to Gόmez, Moraleja, Oliete, Ruiz, and Caballero (2010), crust color is mainly affected by the Maillard reaction rather than type of dietary fiber, whereas crumb color is influenced by the dietary fiber because it does not reach the high temperature necessary to accelerate the Maillard reaction, resulting in brown pigment formation. The browning index decreased with increased KBP, presumably due to the addition of KBP. Matos, Sanz, and Rosell (2014) and Shevkani and Singh (2014) demonstrated that the browning index is highly influenced by the original color of the added ingredients.

The texture (hardness, adhesiveness, springiness, cohesiveness, chewiness, and resilience) of muffins with added KBP was evaluated (Table 4). Textural characteristics are of prime importance since they can affect consumer acceptance of the products (Shevkani & Singh, 2014). The control showed the lowest hardness value (412 N), and as the KBP content increased, the hardness values of the muffins gradually increased. With the addition of KBP, the gluten in the dough was diluted and thereby it might cause decreased gas‐capturing ability and increased density of the muffins. Jung and Cho (2011) noticed similar results in muffins with brown rice flour. The adhesiveness values did not change significantly, corroborating the findings of Im, Kim, and Ha (1998), wherein muffins were supplemented with sorghum flour. The springiness and resilience of muffins with added KBP were significantly lower than in the controls, which again was probably caused by the dilution of gluten. Muffins with more than 2% dietary fiber (KBP 2, 3, and 4) showed significantly lower cohesiveness values than the control muffin; a factor associated with the increased hardness that would result in greater crumbliness than samples with lower KBP levels. The chewiness values of muffins with added KBP were lower than the control values, which was possibly a result of water absorption by the increasing fiber concentration associated with the increased KBP content (Walker et al., 2014).

### 3.3 Sensory evaluation

The scores for product attributes and overall acceptance (Table 5) indicated no significant differences in appearance and color acceptance among all samples. For texture, there was no significant difference up to KBP 2, whereas KBP 3 and 4 had lower values than the others. As KBP was added and muffin hardness increased, the texture acceptance score decreased. The acceptance score for flavor decreased as the ratio of KBP increased, with a significant difference between KBP 3 and 4 compared to the control. For overall acceptance, there was no significant difference between the control, KBP 1 and 2 samples, but the acceptance slightly decreased with the addition of 3% or 4% dietary fiber (KBP 3 and 4). Therefore, the addition of up to 2% dietary fiber (derived from the KBP) was considered to be an appropriate amount, based on the sensory evaluation.

**Table 5:** Sensory evaluation of muffins containing kimchi by‐product powder (KBP)

Attribute	Control	KBP 1^1^	KBP 2	KBP 3	KBP 4
Appearance	4.87 ± 1.38^a^ ^2^	4.24 ± 1.40^b^	4.73 ± 1.14^a^	4.87 ± 1.44^a^	4.92 ± 1.47^a^
Color	4.80 ± 1.38^a^	4.05 ± 1.44^b^	4.66 ± 1.22^a^	5.02 ± 1.53^a^	4.94 ± 1.51^a^
Texture	4.65 ± 1.36^ab^	4.97 ± 1.39^a^	4.73 ± 1.30^ab^	4.56 ± 1.42^b^	4.51 ± 1.48^b^
Flavor	4.72 ± 1.17^a^	4.68 ± 1.41^a^	4.66 ± 1.30^a^	4.30 ± 1.53^b^	3.82 ± 1.47^c^
Overall Acceptance	4.76 ± 1.38^a^	4.82 ± 1.37^a^	4.57 ± 1.36^ab^	4.24 ± 1.51^b^	3.82 ± 1.58^c^

Note

A different superscript letter in the same column indicates statistical difference (*p < *0.05).

^1^ KBP 1, 2, 3, and 4: dietary fiber in KBP replaced 1%, 2%, 3%, and 4% of the flour, respectively.

^2^ All data represented as mean ± standard deviation (*n* = 3).

## 4 CONCLUSION

The results of this study show the possibility of using an abundant kimchi by‐product as a source of dietary fiber. As the amount of KBP increased, the dietary fiber content in the muffins significantly increased compared to that in the control. The height and volume of the muffins decreased with the addition of KBP, while the weight loss rate decreased. With increased KBP, L*, a*, and b* values significantly decreased due to the color of KBP. The addition of KBP showed an increase in hardness due to the weakening of the network structure of gluten. The higher the KBP content, the higher the antioxidant property of muffins. No significant difference was observed in the overall acceptance up to 2% added group, and the positive effect was also confirmed in the sensory evaluation. These results indicate that it is possible to produce functional muffins with increased dietary fiber content by adding KBP in place of flour.

## CONFLICT OF INTEREST

The authors declare that they have no conflict of interest.

## ETHICAL STATEMENTS

This study does not involve any human testing.
